# α_2_-Adrenoreceptor Constraint of Catecholamine Release and Blood Pressure Is Enhanced in Female Spontaneously Hypertensive Rats

**DOI:** 10.3389/fnins.2016.00130

**Published:** 2016-03-30

**Authors:** Torill Berg

**Affiliations:** Division of Physiology, Department of Molecular Medicine, Institute of Basic Medical Sciences, University of OsloOslo, Norway

**Keywords:** α_2_-adrenoceptors, angiotensin AT1 receptor, sympathetic nervous system, norepinephrine, epinephrine, female rats, spontaneously hypertensive rats, total peripheral vascular resistance

## Abstract

α_2_-adrenoceptors (α_2_AR) lower central sympathetic output and peripheral catecholamine release, and may therefore prevent sympathetic hyperactivity and hypertension. The α_2_AR are dysfunctional in male spontaneously hypertensive rats (SHR). Premenopausal females are less hypertensive than males. The purpose of this study was to test if this difference could be explained by functional α_2_AR in the female SHR. A 15-min tyramine-infusion was used to stimulate norepinephrine release through the re-uptake transporter, consequently preventing re-uptake. Presynaptic control of vesicular release will therefore be reflected as differences in overflow to plasma. The surgical trauma activates secretion of epinephrine, also subjected to α_2_AR auto-inhibition. Blood pressure was monitored through a femoral artery catheter and cardiac output by ascending aorta flow in 12-14 weeks-old (early hypertension) SHR and normotensive rats (WKY). Total peripheral vascular resistance (TPR) was calculated. Female SHR, unlike male, were close to normotensive. Pre-treatment with none-selective (clonidine) or non-A-selective (ST-91) α_2_AR agonist reduced, and none-selective α_2_AR antagonist (L-659,066) increased tyramine-induced norepinephrine overflow in female WKY and SHR. L-659,066 also increased secretion of epinephrine. The L-659,066-induced increase in catecholamine release was further enhanced by additional pre-treatment with ST-91 or angiotensin AT1 receptor antagonist (losartan) in SHR only. L-659,066 eliminated the tyramine-induced rise in TPR in both strains in female rats. Conclusion: α_2_AR-mediated control of catecholamine release and vascular tension was therefore functional in female SHR, unlike that previously observed in male SHR. Functional α_2_AR is likely to have a protective function and may explain the lack of hypertension in the young female SHR.

## Introduction

**Figure 1:**
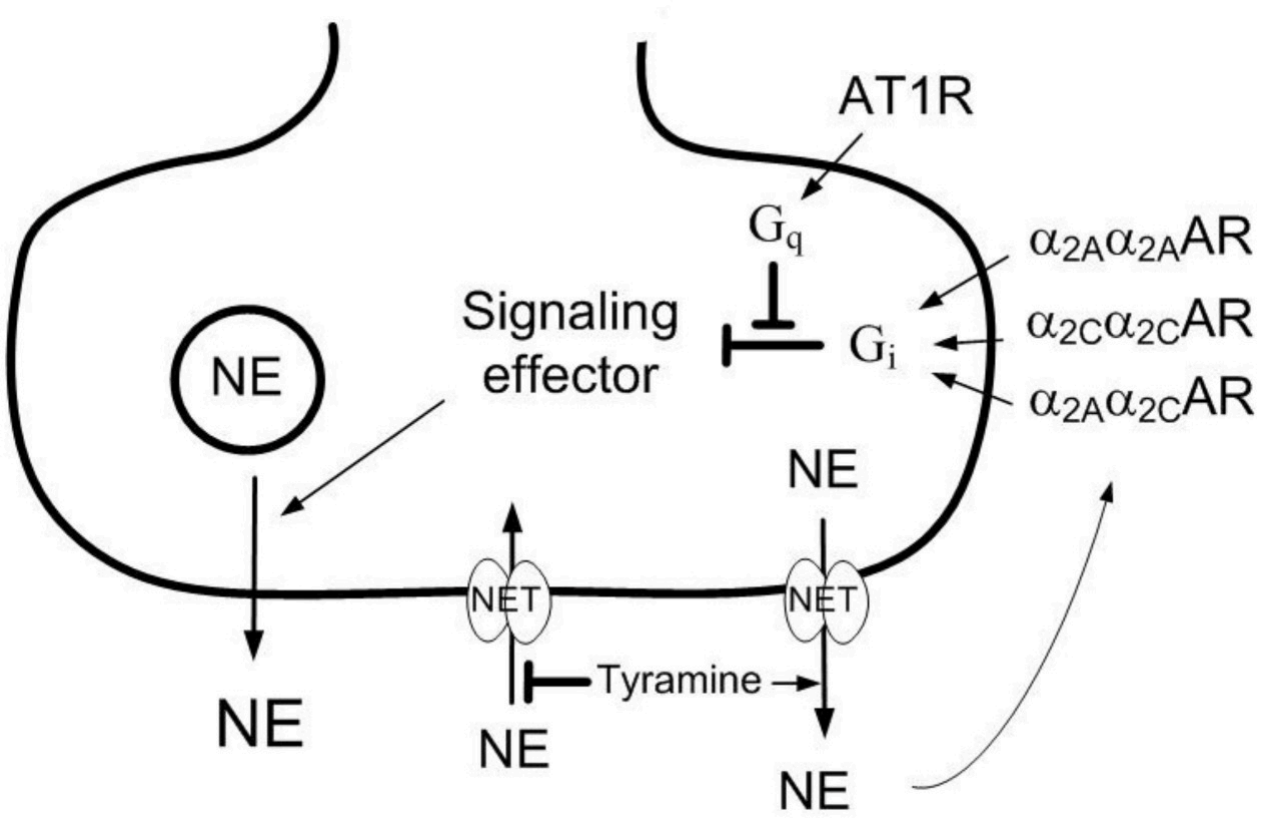
**Presynaptic modulation of vesicular norepinephrine release studied by the use of tyramine**. Tyramine stimulates the release of norepinephrine by reversed transport through the norepinephrine re-uptake transporter (NET). The regular re-up-take of norepinephrine through NET will therefore be prevented, resulting in an elevated overflow to plasma. The released norepinephrine and other agonists present will activate the presynaptic receptors and influence the amount released from synaptic vesicles and, hence, the overflow of norepinephrine (NE) to plasma. Differences in the plasma norepinephrine concentration will therefore reflect changes in release induced by presynaptic receptor agonist or antagonist. Stimulation of the α_2C_AR-subtype may enhance α_2A_AR-subtype-mediated inhibition of release, possibly because the α_2A_α_2C_AR heterodimer is more resistant to G-protein coupled receptor kinase (GRK)-desentization than the α_2A_α_2A_AR or α_2C_α_2C_AR homodimers. The angiotensin AT1 receptor (AT1R) may stimulate release by inhibiting down-stream signaling of the α_2C_AR. However, α_2C_AR may also inhibit renal renin release, and through that reduce presynaptic AT1R counter-action of α_2_ AR auto-inhibition of release. G_q_,G-protein of the q subtype; G_i_,inhibitory G-protein.

Premenopausal women are less hypertensive than males of the same age (Lerner and Kannel, 1986). The same is true in spontaneously hypertensive rats (SHR; Maris et al., 2005). The antihypertensive mechanisms involved are not known (Reckelhoff and Fortepiani, 2004), but a difference in sympathetic activity has been suggested, since the sympathetic tone will increase with age (Ng et al., 1993; Seals and Esler, 2000). The release of catecholamines from sympathetic nerves (Figure 1) and the adrenal glands is inhibited by α_2_-adrenoceptors (AR), and α_2_AR in vascular smooth muscle cells (VSMC) promote vasoconstriction. These functions were failing in male SHR (Remie et al., 1992; Zugck et al., 2003; Berg and Jensen, 2013), and the dysfunctional auto-inhibition of catecholamine release may be a contributory factor in the development of high blood pressure (BP). This conclusion was compatible with the high levels of circulating catecholamines and hypertension observed in α_2A_AR-knock-out mice (Makaritsis et al., 1999). In female rats, α_2_AR antagonist enhanced the release of norepinephrine in response to sympathetic nerve stimulation with a greater effect than in male rats in the isolated heart (Du et al., 1991), although not in the tail artery (Garcia-Villalon et al., 1997). These observations may suggest a gender-related difference in α_2_AR-mediated inhibition of release.

The α_2_AR comprise three subtypes, i.e., A, B, and C. The α_2A_ and α_2C_ are the main subtypes involved in auto-inhibition of catecholamine release, whereas the α_2B_-subtypemayinduce VSMC contraction (Link et al., 1996; Hein et al., 1999; Trendelenburg et al., 2003a; Berg et al., 2012). The failing α_2_AR-mediated inhibition of release in SHR was restored by enhanced α_2C_AR signaling, achieved either directly by α_2C_AR agonist, or indirectly by angiotensin AT1 receptor (AT1R) antagonist (Berg, 2013). The latter may be explained by that angiotensin II, through the AT1R, stimulated release by interfering with inhibitory G (G_i_)-protein signaling (Figure 1), an interaction which involved the α_2C_-subtype only (Trendelenburg et al., 2003b). Since the renin-angiotensin system has been implicated in postmenopausal hypertension (Reckelhoff and Fortepiani, 2004), the impact of α_2C_AR and AT1R on α_2_AR function may be different in male and female SHR.

The present study therefore analyzed if there was a gender-related difference in α_2_AR auto-inhibition of catecholamine release and/or control of vascular tension which may explain the lower BP in the female SHR. α_2_AR functionality was studied by the effect of the non-selective α_2_AR antagonist L-659,066 on tyramine-stimulated norepinephrine release. Tyramine stimulates norepinephrine release by reversing the transport through the norepinephrine re-uptake transporter (NET), consequently blocking re-uptake (Figure 1). Activation of the presynaptic receptors by the released norepinephrine and/or other agonists present will alter the vesicular release of norepinephrine, and this will be reflected as differences in the overflow of norepinephrine to plasma (Berg et al., 2012; Berg and Jensen, 2013). Neither L-659,066, nor tyramine, crosses the blood-brain barrier (Oldendorf, 1971; Clineschmidt et al., 1988). The tyramine-induced release of norepinephrine is therefore not directly dependent on the sympathetic tone, which will be influenced by factors such anesthesia, ventilation and rat strain, but activated pharmacologically in the nerve terminal. Tyramine also allowed a simultaneous study of the role of α_2_AR in the cardiovascular response to norepinephrine release. Furthermore, as established in male rats, the trauma induced by the surgical procedure activated some secretion of epinephrine, also subjected to receptor-mediated release control (Berg et al., 2012; Berg and Jensen, 2013).

## Materials and methods

### Experimental procedure

All experiments were approved by The Norwegian Animal Research Authority (NARA) and conducted in accordance with the Directive 2010/63/EU of the European Parliament. Normotensive rats (Wistar Kyoto, WKY) and SHR (Okamoto, SHR/NHsd strain) were bred in-house, and the female rats included in this study were from the same litters as male rats included here and in previous publications. The rats used in the experimental protocols below were 12–14 weeks old, representing the early hypertensive stage in male SHR. Forty-nine female WKY (179 ± 2 g b.w.) and 8 male WKY (291 ± 9 g b.w.), and 48 female SHR (178 ± 1 g b.w.) and 8 male SHR (276 ± 13 g b.w.) were included. In addition, BP was recorded in anesthetized, retired breeders (6–8 rats per group) from the same stock (245 ± 8 and 391 ± 7 g b.w., 49 ± 1 and 45 ± 1 weeks, respectively, in female and male WKY, and 231 ± 7 and 401 ± 10 g b.w., 54 ± 3 and 47 ± 3 weeks, in female and male SHR). The older rats were not subjected to further experiments. The rats were housed on 12/12 h light/dark cycles, and allowed food (conventional rat chow diet with 0.7% NaCl) and water *ad lib* until the time of the experiment. The rats were anesthetized with pentobarbiturate (70–75 mg/kg, i.p.) and tracheotomised. Systolic (SBP) and diastolic (DBP) BP were monitored through a catheter in the femoral artery, which also recorded heart rate (HR) before the rats were connected to ventilator. After thoracotomy, entering through the third intercostal space, cardiac output (CO = minus cardiac flow) and HR were recorded by a flow probe on the ascending aorta, connected to a T206 Transonic Flow meter (Transonic Systems Inc., Ithaka NY, USA). Mean arterial BP (MBP = SBP-DBP/3+DBP) and total peripheral vascular resistance (TPR = MBP/CO) were calculated. The rats were on a positive pressure ventilator throughout the experiment, ventilated with air. Body temperature was maintained at 37–38°C by external heating, guided by a thermo sensor inserted inguinally into the abdominal cavity. All drugs were dissolved in PBS, and injected through a catheter in the femoral vein (0.6–1.0 ml/kg). When all surgery was completed, the arterial catheter was flushed with 0.1 ml PBS (0.01 M Na-phosphate, pH 7.4, 0.14 M NaCl) containing 500 IU heparin/ml. The rats were then allowed to stabilize for 10 min.

### Experimental design

**Figure 2:**
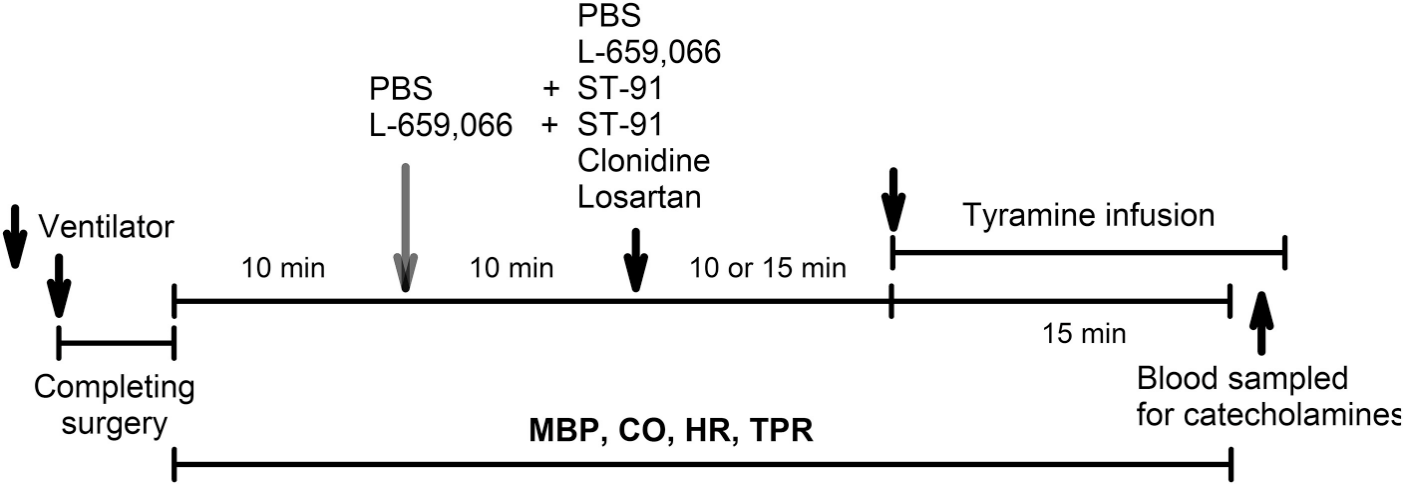
**An overview of the design of the experiments performed on 12–14 weeks-old rats**. PBS, sham injection with vehicle; L-659,066, peripherally restricted, non-selective α_2_AR-antagonist; Clonidine, not restricted, non-selective α_2_AR-agonist; ST-91, peripherally restricted α_2(non−A)_AR-agonist; Losartan, AT1R antagonist; Tyramine, stimulates reverse transport of norepinephrine through the re-uptake transporter.

An overview of the experiments performed on the 12–14 weeks-old rats is shown in Figure 2. All rats were infused with tyramine (1.26 μmol/min/kg, 15 min) to stimulate endogenous norepinephrine release (Berg et al., 2012; Berg and Jensen, 2013). Control rats were pre-treated with PBS 10 min before tyramine. The experimental groups were pre-treated with the α_2_AR-antagonist L-659,066 (4.4 μmol/kg, –10 min) (Berg et al., 2012; Berg and Jensen, 2013), with PBS followed 10 min later by α_2(non−A)_AR-agonist ST-91 (24 nmol/kg) (Berg, 2013), or with L-659,066 followed by ST-91, allowing 15 min between ST-91 and tyramine. These drugs do not cross the blood-brain barrier (Oldendorf, 1971; Clineschmidt et al., 1988; Takano et al., 1992). Rats were also pre-treated with the non-selective α_2_AR-agonist clonidine (151 nmol/kg) (Berg and Jensen, 2013), which crosses the blood-brain barrier, followed by tyramine 15 min later. In addition, rats were pre-treated with the AT1R antagonist losartan (79 μmol/kg) (Berg, 2002), followed by PBS or L-659,066 10 min later. Six-8 rats were included in each group.

### Measurement of plasma catecholamines

Blood was sampled from the arterial catheter after the tyramine-observation period, but without discontinuing the tyramine infusion. The blood (1.5 ml) was collected into tubes containing 40 μl 0.2 M glutathione and 0.2 mol/L ethylene glycol-bis (2-aminoethylether)-N,N,N',N'-tetraacetic acid (EGTA) (4°C). Plasma was stored at –80°C until catecholamine concentrations were determined using 400 μl plasma and the 5000. Reagent kit for HPLC analysis of Catecholamines in plasma from Chromsystems GmbH, Munich, Germany, as described by the manufacturer. The samples were run on a Shimadzu monoamines analyzer (HPLC) system, using an isocratic flow rate of 0.8 ml/min, and an electrochemical detector (Decade II) and a SenCell electrochemical flow cell (Antec Leyden, Zoeterwoude, The Netherlands).

### Drugs

L-659,066 was a kind gift from Merck, Sharp and Dohme Labs, Rahway, NJ, USA. ST-91 was from TOCRIS bioscience, Bristol, UK. The remaining drugs were from Sigma Chemical Co., St. Louis, MO, USA.

### Statistical analyses

Results are presented as mean values ± s.e.mean. Data were averaged every min in all experiments. For the narrow peak-pressor response to ST-91, data were averaged every 5 s. The cardiovascular response-curves to agonists and tyramine were analyzed using Repeated Measures Analyses of Variance and Covariance, first as over-all tests within each strain, and subsequently for each group separately or between groups. Significant responses were subsequently located at specific times using one-sample Student's *t*-tests, and were indicated in the figures with an asterix within the group symbol. Differences between groups at the same times were identified using two-sample Student's *t*-tests. For the TPR-response, these *ad hoc* analyses were done for (1) the TPR-peak-response which occurred after 3 or 4 min, or at 4 min when a TPR-peak was not present, and (2) after 15 min. For the HR-response, the Student's *t*-tests were performed only at 15 min. The plasma catecholamine concentrations were first analyzed using two-way ANOVA, and the cardiovascular baselines and the effect of pre-treatment by one-way ANOVA. Group and strain-related differences were subsequently located by two-sample Student's *t*-tests for parametric data, or, in the presence of out-liars, by nonparametric Kruskal-Wallis tests. For all analyses, testing proceeded only when significant responses, differences and/or interactions were indicated. The *P*-value was for all tests and each step adjusted according to Bonferroni, except for the catecholamine data, where *P* ≤ 0.05 was considered significant. The number of rats included in each group, i.e., 6–8 rats, was based on sample power calculations using previous data from similar or related experiments.

## Results

### The influence of α_2_AR and AT1R on the plasma catecholamine concentrations (Table 1)

#### Norepinephrine

The tyramine-induced norepinephrine overflow to plasma in female control rats of both strains was not different from that in male rats (*P* = NS). The concentration of norepinephrine was greater in SHR than in WKY in both genders (*P* = 0.002). In females of both strains, the non-selective α_2_AR agonist clonidine reduced the tyramine-induced norepinephrine overflow to plasma (*P* ≤ 0.022), whereas the antagonist L-659,066 increased norepinephrine overflow (*P* = 0.044 and *P* = 0.007 in WKY and SHR, respectively). The α_2(non−A)_AR agonist ST-91 and the AT1R antagonist losartan had alone no effect on the tyramine-induced overflow in either strain (*P* = NS). After L-659,066+ST-91+tyramine, norepinephrine overflow was not different from that after L-659,066+tyramine in female WKY (*P* = NS), but was higher in SHR (*P* = 0.015). Overflow to plasma was higher in female WKY pre-treated with losartan+L-659,066 than that in the controls (*P* = 0.039) but not different from that after losartan alone (*P* = NS) and slightly less than that after L-659,066 alone (*P* = 0.003). In female SHR, losartan potentiated the augmenting effect of L-659,066 on tyramine-induced norepinephrine overflow (*P* ≤ 0.006 compared to the controls or the losartan/L-659,066-only groups). Please see (Berg, 2013; Berg and Jensen, 2013) for similar studies on male rats.

**Table 1:** **The plasma concentration of norepinephrine and epinephrine at the end of the tyramine-infusion period in 12–14 weeks old rats**.

	**WKY**		**SHR**	
	**Norepinephrine (nM)**	**Epinephrine (nM)**	**Norepinephrine (nM)**	**Epinephrine (nM)**
***MALE RATS:***				
PBS+tyramine	21.0 ± 1.2	4.3 ± 1.3	27.3 ± 2.0^*^	6.7 ± 1.1
***FEMALE RATS:***				
PBS+tyramine	23.4 ± 1.6	1.5 ± 0.7	32.7 ± 1.9^*^	4.1 ± 2.2
Clonidine+tyramine	18.0 ± 0.5^†^	1.2 ± 0.3	27.1 ± 0.8^*^^†^	1.8 ± 0.5
L-659,066+tyramine	34.4 ± 4.2^†^	15.3 ± 5.7^†^	43.9 ± 2.8^†^	11.2 ± 3.1^†^
PBS+ST-91+tyramine	25.7 ± 2.1	3.6 ± 1.5	36.5 ± 3.8^*^	10.5 ± 5.4
L-659,066+ST-91+tyramine	36.1 ± 3.4^†^^‡^	15.2 ± 4.3^†^^‡^	53.6 ± 2.9^*^^†^^‡^§	21.3 ± 2.7^*^^†^^‡^§
Losartan+PBS	25.3 ± 0.7	6.4 ± 1.7^†^	27.1 ± 1.9	3.7 ± 1.3
Losartan+L-659,066+tyramine	30.3 ± 2.4^†^§	10.2 ± 2.5^†^	67.7 ± 9.0^*^^†^^‡^§	27.0 ± 5.3^†^^‡^§

Differences were detected as indicated between corresponding SHR and WKY groups (^*^ after SHR values), between the female PBS+tyramine controls and corresponding experimental groups (^†^), between corresponding groups pre-treated with ST-91/losartan alone and ST-91/losartan+L-659,066 (^‡^), and between the groups pre-treated with ST-91/losartan+L-659,066 and L-659,066 alone (§). Gender-dependant differences within each strain in rats treated with PBS+tyramine were not detected. In male time-controls, infused with PBS instead of tyramine, the plasma norepinephrine and epinephrine concentrations were 0.7 ± 0.2 and 6.9 ± 1.2 nM, respectively in WKY, and 1.3 ± 0.2 and 10.9 ± 1.9 nM in SHR (Berg, 2014).

*, †, ‡ P ≤ 0.05.

#### Epinephrine

Strain- and gender-related differences were not observed in the plasma epinephrine concentration. Clonidine, ST-91 and losartan had no effect on the plasma epinephrine concentration in female rats of either strain (*P* = NS), except for a small increase after losartan in WKY (*P* = 0.037). The antagonist L-659,066 increased the secretion of epinephrine in both strains (*P* ≤ 0.021). The plasma epinephrine concentration after ST-91/losartan+L-659,066 was not different from that after L-659,066 alone in WKY, but was higher than that after L-659,066-only in SHR (*P* ≤ 0.036).

### Cardiovascular baselines and response to pre-treatment

#### Strain-, gender- and age-related differences in BP and HR

When BP and HR were recorded in the anesthetized rats after femoral artery catheterization but before the rats were connected to the respirator (Table 2), the 12–14 weeks-old male SHR were clearly hypertensive whereas the female SHR of the same age were not. Also baseline HR was higher in these SHR than in WKY in the males (*P* < 0.001) but not in the females (*P* = NS). Thus, HR was higher in male than in female SHR (*P* < 0.001), whereas a gender-dependant difference was not present in WKY (*P* = NS). However, when the rats were about 1 year old, both male and female SHR were hypertensive (*P* ≤ 0.007) and HR was higher than that in WKY in both genders (*P* = 0.001; Table 2). Age-related differences in BP and HR were not observed in WKY. These observations demonstrated that the female SHR were in a pre-hypertensive stage at the age of 12–14 weeks.

**Table 2:** **Resting BP and HR in young and old, male and female WKY and SHR**.

**WKY**					**SHR**			
	**SBP mm Hg**	**DBP mm Hg**	**MBP mm Hg**	**HR bpm**	**SBP mm Hg**	**DBP mm Hg**	**MBP mm Hg**	**HR bpm**
**12–14 WEEKS OLD RATS**									
Male	103 ± 8	73 ± 6	83 ± 6	312 ± 15	183 ± 18^*^	146 ± 12^*^	157 ± 15^*^	421 ± 9^*^
Female	87 ± 7	61 ± 7	70 ± 7	317 ± 13	108 ± 8^†^	75 ± 8^†^	86 ± 8^†^	349 ± 10^†^
**45–54 WEEKS OLD RATS**									
Male	109 ± 4	76 ± 8	87 ± 7	253 ± 20	175 ± 12^*^	140 ± 10^*^	152 ± 10^*^	367 ± 14^*^^‡^
Female	96 ± 9	68 ± 7	78 ± 8	285 ± 5	170 ± 14^*^^‡^	129 ± 11^*^^‡^	143 ± 12^*^^‡^	376 ± 16^*^

BP and HR baselines were recorded through the femoral artery before the rats were connected to the respirator and before thoracotomy. Comparisons were made between WKY and SHR within each gender (* after SHR values), between male and female rats within each strain (^†^ after female values) and between corresponding young and older rats (^‡^ after older rats). MBP in both strains and HR in WKY were reduced by the anesthesia, and were in chronically catheterized, awake, 12-14 weeks-old rats measured to be 113 ± 8 and 169 ± 4 mm Hg, and 399 ± 22 and 407 ± 12 bpm, in male WKY and SHR, respectively. SBP, systolic blood pressure; DBP, diastolic blood pressure; MBP, mean arterial blood pressure; HR, heart rate.

*, † P ≤ 0.009.

#### Impact of artificial ventilation on BP

As previously discussed for male rats (Berg and Jensen, 2013), SHR were more sensitive to the reduced venous return to the right heart during positive-PEEP-ventilation than WKY also in female rats. Thus, after the 12–14 weeks-old rats were connected to the respirator and surgery completed, i.e., prior to pre-treatment, SBP/DBP/MBP were reduced in male SHR and SBP in female SHR (*P* ≤ 0.008), but not in male or female WKY (*P* = NS; Table 3). The changes in HR were not statistically significant.

**Table 3:** **Cardiovascular baselines in the 12–14 weeks old rats after pre-treatment, i.e., prior to tyramine, with the response to pre-treatment shown below in parenthesis**.

**Pre-treatment**	**WKY**				**SHR**			
	**MBP mm Hg**	**HR bpm**	**CO ml/min**	**TPR mm Hg/ml/min**	**MBP mm Hg**	**HR bpm**	**CO ml/min**	**TPR mm Hg/ml/min**
***Male:***								
PBS	61 ± 4 (−8 ± 3)	330 ± 14 (-12 ± 6)	32 ± 2 (0 ± 1)	1.9 ± 0.1 (−0.3 ± 0.1)	92 ± 7^*^ (−6 ± 3)	399 ± 10^*^ (−4 ± 4)	22 ± 2^*^ (−1 ± 1)	4.3 ± 0.3^*^ (−0.2 ± 0.2)
***Female:***								
PBS	75 ± 5 (1 ± 3)	344 ± 8 (−1 ± 5)	26 ± 0^†^ (1 ± 1)	2.9 ± 0.2^†^ (−0.1 ± 0.1)	56 ± 3^*^^†^ (−10 ± 3)	366 ± 11 (−25 ± 3)	16 ± 1^*^ (−1 ± 0)	3.6 ± 0.1^*^ (−0.5 ± 0.2)
Clonidine	64 ± 5 (−2 ± 3)	314 ± 16 (−29 ± 8)	26 ± 2 (6 ± 1)^‡^	2.6 ± 0.3 (−0.8 ± 0.2)^‡^	55 ± 3 (−18 ± 8)	313 ± 14 (−81 ± 11)^‡^	16 ± 2 (3 ± 1)	3.6 ± 0.3 (−2.0 ± 0.3)^‡^
L-659,066	54 ± 6 (−8 ± 3)	356 ± 8 (−4 ± 5)	26 ± 3 (0 ± 1)	2.3 ± 0.3 (−0.2 ± 0.1)	52 ± 4 (−25 ± 4)	350 ± 11 (−40 ± 8)	12 ± 1 (−4 ± 1)	5.3 ± 1.5 (0.2 ± 0.9)
PBS+ST-91	76 ± 9 (12 ± 10)	352 ± 15 (−11 ± 4)	29 ± 2 (3 ± 1)	2.8 ± 0.4 (0.2 ± 0.3)	97 ± 15 (28 ± 3)	348 ± 14 (−64 ± 18)	16 ± 3 (0 ± 1)	6.7 ± 1.4 (2.0 ± 1.0)
L-659,066+ST-91	54 ± 2 (−14 ± 6)	349 ± 11 (−17 ± 18)	30 ± 2 (5 ± 2)	1.8 ± 0.1^‡^ (−0.9 ± 0.3)^‡^	82 ± 12 (4 ± 3)	362 ± 6 (−41 ± 11)	20 ± 6 (−1 ± 1)	5.6 ± 1.3 (1.1 ± 0.8)
Losartan	40 ± 2 (−29 ± 4)^‡^	345 ± 11 (−25 ± 7)	24 ± 1 (−5 ± 2)^‡^	1.7 ± 0.1^‡^ (−0.7 ± 0.2)	48 ± 3^‡^ (−38 ± 6)^‡^	325 ± 7 (−62 ± 4)^‡^	13 ± 1 (−3 ± 0)^‡^	3.8 ± 0.2 (−1.7 ± 0.3)^‡^
Losartan+L-659,066	37 ± 2 (−29 ± 4)^‡^	327 ± 14 (−25 ± 10)	26 ± 1 (0 ± 1)	1.4 ± 0.1 (−1.1 ± 0.1)^‡^	37 ± 2^‡^ (−41 ± 8)^‡^	341 ± 7 (−48 ± 11)^‡^	12 ± 1 (−3 ± 1)	3.3 ± 0.2 (−2.2 ± 0.7)^‡^

Comparisons were made between the WKY and SHR controls within each gender (* after SHR values), between female and male PBS-controls within each strain (^†^ after female values), and between the female PBS-controls and the experimental groups (^‡^). Significant differences were not detected between the groups pre-treated with PBS+ST-91 (α_2C_AR-subtype-selective agonist) and L-659,066 (peripheral, nonselective α_2_AR antagonist) + ST-91, or between the groups pre-treated with the angiotensin AT1 receptor antagonist losartan alone and losartan+L-659,066 (P < 0.025). MBP, mean arterial blood pressure; HR, heart rate; CO, cardiac output; TPR, total peripheral vascular resistance.

*, † P ≤ 0.0125,

‡ P ≤ 0.0083.

#### Strain- and gender-related differences in CO and TPR

CO and TPR were monitored only after the 12–14 weeks-old rats were connected to the computer, and the surgery was completed. CO baseline was higher in male than in female rats of both strains (Table 3), but when adjusted for the gender-dependant difference in body weight, the cardiac index (CI = CO/kg body weight) was higher in females than in males in WKY (142 ± 5 and 110 ± 6 ml/min/kg, respectively, *P* = 0.001), but not different in SHR (90 ± 5 and 78 ± 6 ml/min/kg, *P* = NS). A lower CI was therefore observed in SHR compared to WKY in both genders (*P* ≤ 0.005). At this time, baseline TPR was higher in SHR than in WKY in the males (*P* = 0.009), but not different in the females (*P* = NS; Table 3).

#### The effect of pre-treatment

**Figure 3:**
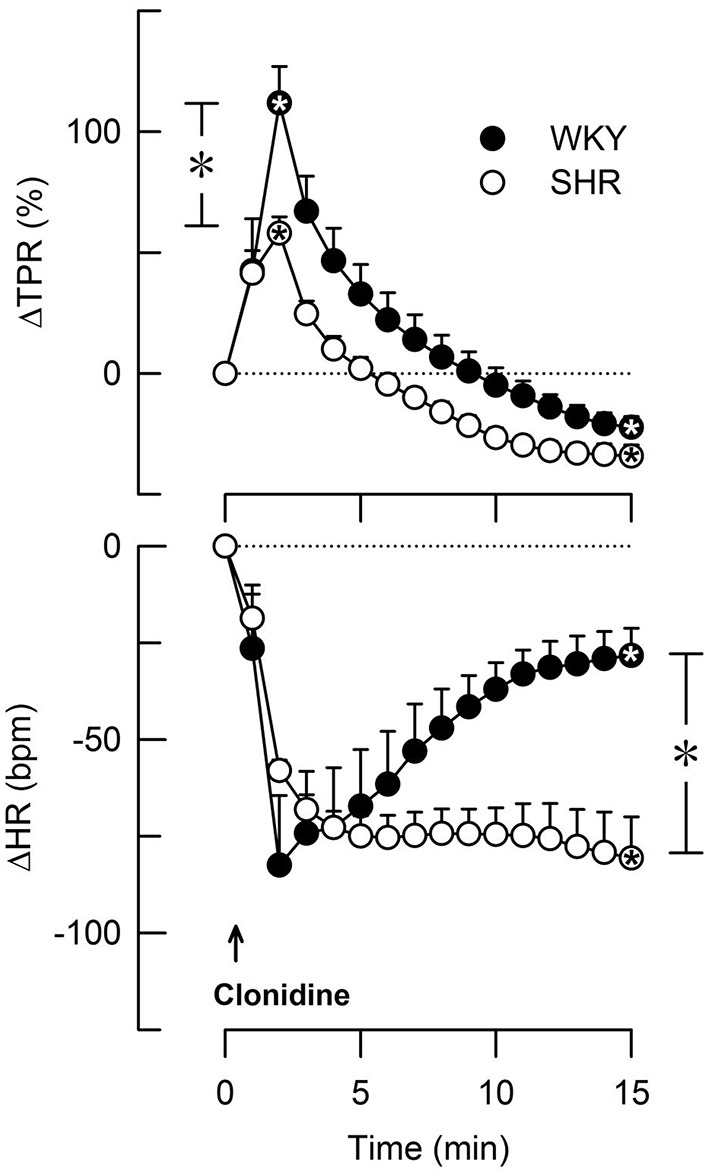
**The change in total peripheral vascular resistance (TPR) and heart rate (HR) in response to the non-selective α_2_AR agonist clonidine in female WKY and SHR (12–14 weeks-old rats)**. Clonidine penetrates the blood-brain barrier. TPR prior to clonidine was 2.6 ± 0.3 and 3.6 ± 0.3 mm Hg/ml/min in WKY and SHR, respectively (*P* = 0.02). *Within symbols; significant responses, and * in brackets; significant differences between WKY and SHR, detected at peak/nadir response or after 15 min, as indicated. ^*^, ^*^P < 0.025 after curve evaluation.

Group differences in baseline MBP, HR, CO and TPR prior to pre-treatment within each strain of female rats were not observed (*P* = NS). Baselines after pre-treatment and the effect of pre-treatment in the 12–14 weeks-old rats are shown in Table 3. Clonidine induced an immediate and transient rise in BP and TPR, which subsequently declined to below baseline in both strains (*P* ≤ 0.009 after 15 min; Figure 3). Also ST-91 (not shown) induced a transient and highly variable (51–825%) rise in TPR in the female rats of both strains, and a significant reduction was not seen after prior administration of L-659,066. Large variations were observed in the TPR-response to ST-91 also after 15 min, and an effect of L-659,066 could not be deciphered (Table 3). Both clonidine (Figure 3) and ST-91 induced a transient bradycardia in WKY, whereas clonidine precipitated a sustained reduction in HR in SHR (*P* = 0.004 for WKY vs. SHR after 15 min). L-659,066 eliminated the immediate HR-response to ST-91 in WKY (*P* = 0.025 compared to the ST-91-only group). L-659,066 had no effect on the cardiovascular baselines, whereas losartan reduced MBP and TPR in both strains and in SHR also HR (*P* ≤ 0.007; Table 3). The effect of losartan+L-659,066 was largely the same as that of losartan alone, although the differences were not always statistically significant.

### The influence of α_2_AR- and AT1R on the cardiovascular response to tyramine in 12–14 weeks-old rats

#### The cardiovascular response to tyramine in female and male control rats

**Figure 4:**
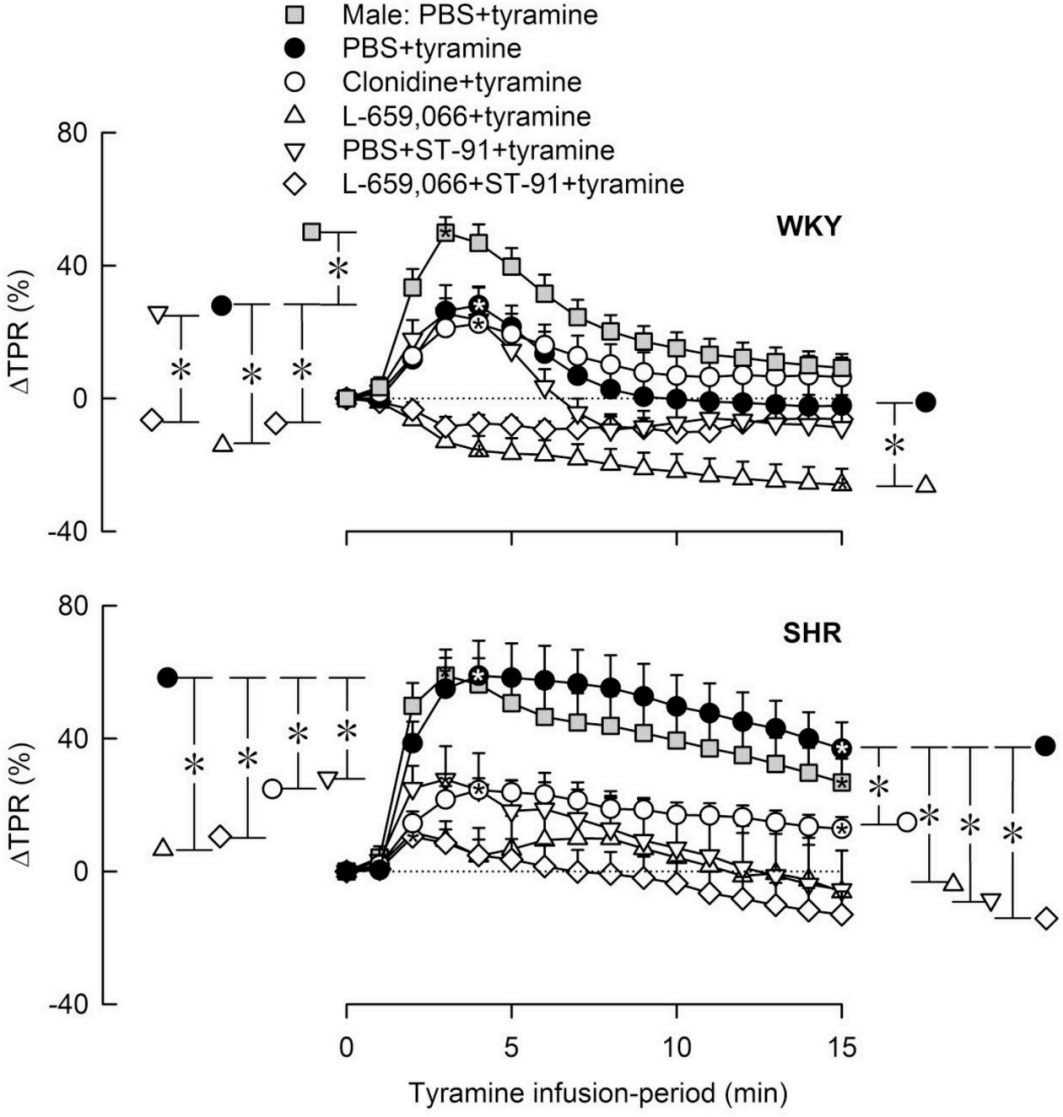
**The change in total peripheral vascular resistance (TPR) in response to tyramine-induced norepinephrine release in 12–14 weeks-old, male and female WKY and SHR control rats, and in female rats pre-treated with α_2_AR agonist and/or antagonist as indicated by the symbol legends**. Baselines prior to tyramine are shown in Table 3. Significant responses (^*^ within symbol) and differences between corresponding male and female control groups, between the female control and experimental groups, and between corresponding PBS+ST-91+tyramine and L-659,066+ST-91+tyramine groups were located at peak response, or after 4 min when a peak was not present (^*^, brackets left of curves). Similar analyses were done after 15 min (^*^, brackets right of curves). Significant responses and group differences other than those indicated were not found. The TPR-peak-response is shown as a bar graph in Figure 5. ^*^, ^*^P ≤ 0.025 after curve evaluations.

**Figure 5:**
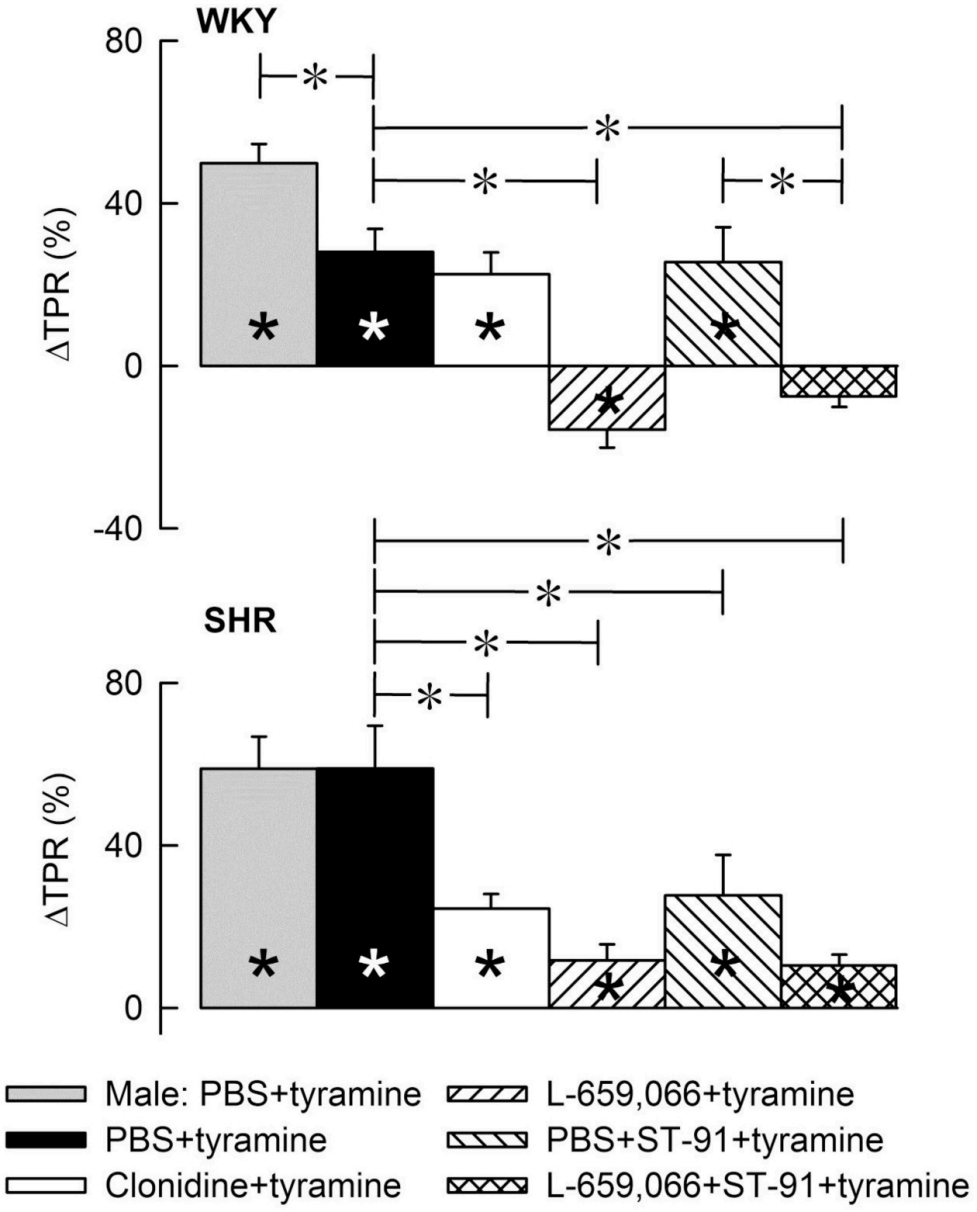
**A bar graph demonstrating the initial change in total peripheral vascular resistance (TPR) in response to tyramine in the same experiments as shown in Figure 4.** Group identity is indicated by the symbol legends, i.e., male controls (PBS+tyramine) and female controls and experimental groups pre-treated with α_2_AR agonist or antagonist. Significant responses (^*^ within column) and differences between corresponding male and female control groups, between the female control and experimental groups, and between corresponding groups given PBS+ST-91+tyramine and L-659,066+ST-91+tyramine were located at peak response, or after 4 min when a peak was not present (^*^, brackets left of curves). Statistical analyses were performed as indicated in Figure 4 legend. ^*^, ^*^P ≤ 0.025 after curve evaluations.

**Figure 6:**
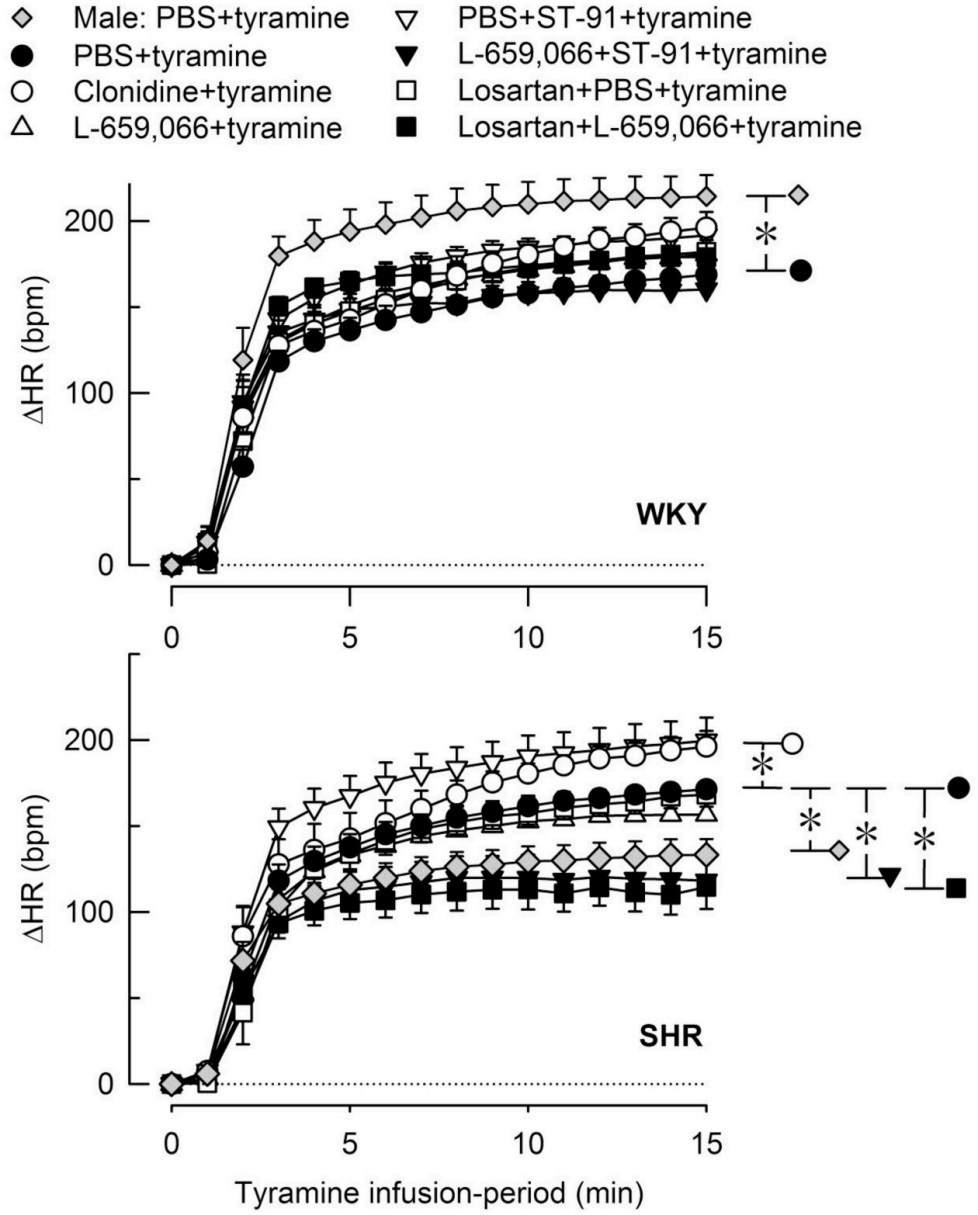
**The heart rate (HR)-response to tyramine-induced norepinephrine release in 12–14 weeks-old, male and female WKY and SHR control rats, and in female rats pre-treated with α_2_AR agonist and/or antagonist as indicated by the symbol legends**. Baselines prior to tyramine are shown in Table 3. The change in HR after 15 min was statistically significant in all groups (not indicated). Differences between the corresponding male and female control groups, and between the corresponding female control and experimental groups were detected as indicated at 15 min (^*^, brackets right of curves). ^*^P ≤ 0.05 after curve evaluations.

Like in male rats, tyramine induced a rise in TPR also in female rats (Figures 4, 5) and a sustained increase in MBP, CO (not shown) and HR (Figure 6). As in the male, the TPR-response in female rats was transient in WKY, but sustained in SHR (*P* = 0.004 for a strain-related difference at 15 min; Figure 4). The TPR peak-response was less in females than in males in WKY (*P* < 0.001) but not different in SHR (*P* = NS; Figures 4, 5). Also the parallel MBP-peak response to tyramine was slightly less in the females than in the males in WKY (ΔMBP at 5 min = 47 ± 3 and 60 ± 3 mm Hg, respectively, *P* = 0.006), but higher in the females in SHR (67 ± 6 and 51 ± 7 mm Hg, *P* = 0.003). The MBP peak-response was higher in SHR than in WKY in the females only (*P* = 0.013). However, the rise in CO at the end of the 15 min-tyramine-infusion period, expressed in percentage change to correct for differences in body weight and baseline CI, was about half in male SHR compared to that in females (ΔCO = 27 ± 5 and 56 ± 7%, respectively, *P* = 0.008), but not different in WKY (64 ± 8 and 60 ± 1% in male and females, respectively, *P* = NS). In the males, the tyramine-induced tachycardia in SHR was about half of that in WKY (*P* = 0.001), whereas a strain-related difference was not observed in the females (*P* = NS; Figure 6).

#### The effect of α_2_AR agonist and antagonist on the TPR-response to tyramine in female rats

Pre-treatment with clonidine or ST-91 had no effect on the TPR-response to tyramine in female WKY (*P* = NS), whereas L-659,066 changed the vasoconstriction to a vasodilatory response (Figures 4, 5). Additional pre-treatment with ST-91 eliminated the late part of this vasodilatation. In female SHR, clonidine reduced, but did not eliminate, the vasoconstrictory TPR-response to tyramine (Figures 4, 5). Also ST-91 reduced the TPR-peak-response and in addition eliminated the late vasoconstriction induced by tyramine in this strain. Furthermore, L-659,066, alone or combined with ST-91, almost totally eliminated the vasoconstriction throughout the infusion-period in the female SHR, although did not induce vasodilatation as in WKY (Figures 4, 5).

#### The interaction between AT1R and α_2_AR in the TPR-response to tyramine in female rats

**Figure 7:**
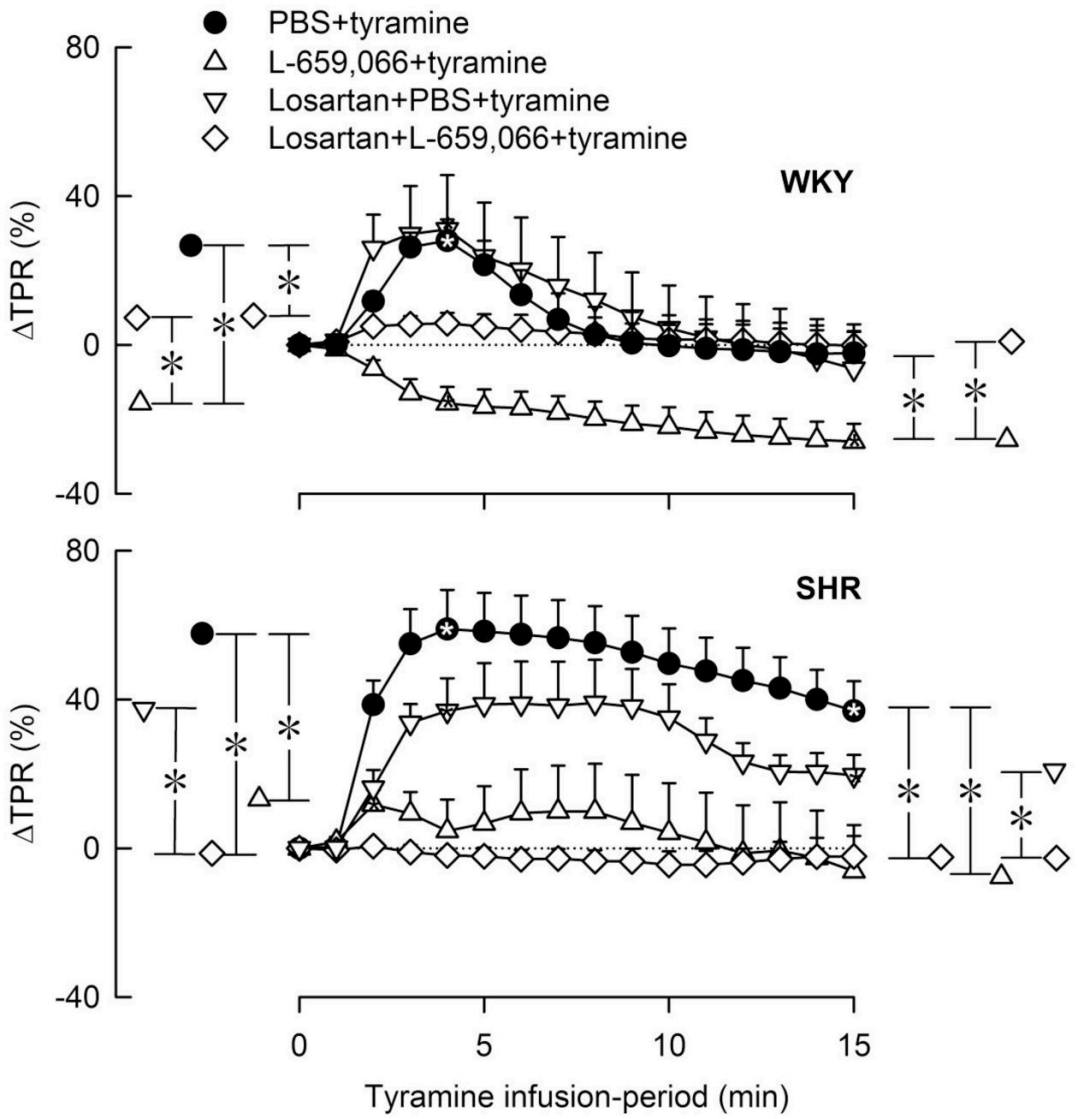
**The change in total peripheral vascular resistance (TPR) in response to tyramine in 12–14 weeks-old, female WKY and SHR pre-treated with the AT1R antagonist losartan, alone or combined with L-659,066**. Baselines prior to tyramine are shown in Table 3. Significant responses (^*^ within symbol) and group differences between the control and experimental groups, and between the losartan+L-659,066+tyramine and the L-659,066+tyramine or losartan+tyramine groups were detected at peak response, or after 4 min when a peak was not present (^*^, brackets left of curves). Similar analyses were performed for the response after 15 min (^*^, brackets right of curves) as indicated. ^*^, ^*^P ≤ 0.025 after curve evaluations.

Losartan alone had no significant effect on the TPR-response to tyramine in either strain in female rats (Figure 7). In WKY, losartan eliminated the L-659,066-dependant vasodilatory response to tyramine. In SHR, the TPR-response to tyramine was eliminated after pre-treatment with losartan+L-659,066, as after L-659,066 alone (Figure 7).

#### The influence of α_2_AR and AT1R on the HR-response to tyramine in female rats

None of the pre-treatments influenced the tyramine-induced tachycardia in the female WKY (Figure 6). In the female SHR, there was a slight increase after clonidine and a slight reduction after L-659,066+ST-91 and losartan+L-659,066. L-659,066 and losartan alone did not influence the tachycardia (Figure 6).

## Discussion

The main findings in the present study were that α_2_AR inhibition of peripheral norepinephrine and epinephrine release and control of vascular tension during tyramine-stimulated norepinephrine release was functional in the anesthetized, 12–14 weeks-old, female SHR, unlike that previously observed in age-matched male SHR (Berg and Jensen, 2013). Although the female SHR did develop high BP later in life, at this early age, the female SHR were pre-hypertensive with a resting BP and HR close to that in the female WKY and much lower than that in the male SHR.

### The role of α_2_AR in control of catecholamine release in 12–14 weeks-old rats

The ability of presynaptic α_2_AR to limit peripheral norepinephrine release in female rats was demonstrated by the fact that the non-selective α_2_AR-agonist clonidine reduced tyramine-induced norepinephrine overflow to plasma in both strains. In the males, clonidine reduced overflow only in SHR, indicating that α_2_AR were fully activated in the male WKY but could be further activated in male SHR (Berg and Jensen, 2013). Although clonidine easily crosses the blood-brain barrier, tyramine does not. The differences in norepinephrine overflow after clonidine were therefore likely to depend on activation of presynaptic α_2_AR in peripheral sympathetic nerves. The peripherally-acting α_2(noneA)_AR agonist ST-91 did not lower tyramine-induced norepinephrine overflow in female rats, suggesting that auto-inhibition of release depended primarily on the α_2A_AR subtype, similar to that previously observed in male rats (Starke, 2001; Brede et al., 2004; Berg, 2013).

However, the adrenal α_2_AR in females seemed almost fully activated in both strains since the clonidine-induced reduction in the plasma epinephrine concentration was not statistically significant in either strain. Clonidine reduced the plasma epinephrine concentration in both strains in the males (Berg and Jensen, 2013). Adrenal epinephrine secretion may therefore be better controlled by α_2_AR in female than in male SHR.

An actual contribution of the α_2_AR in inhibition of release was confirmed by the increased plasma catecholamine concentrations after pre-treatment with the non-selective α_2_AR-antagonist L-659,066. Different from that in the males, where L-649,066 increased tyramine-induced norepinephrine overflow in WKY only (Berg and Jensen, 2013), L-659,066 increased the plasma norepinephrine concentration in both strains in female rats. Also the α_2_AR-mediated auto-inhibition of epinephrine secretion was functional in both strains in female rats, demonstrated by an increased plasma epinephrine concentration after pre-treatment with L-659,066. A similar increase was seen only in WKY and not in SHR in male rats (Berg and Jensen, 2013). Thus, in the female, pre-hypertensive SHR, α_2_AR-mediated auto-inhibition of norepinephrine and epinephrine release was clearly functional, unlike that in the male SHR.

The failing α_2A_AR-mediated inhibition of catecholamine release in male SHR was restored by augmented α_2C_AR signaling, either by directly stimulating the α_2C_AR with agonist or by inhibiting the angiotensin AT1R (Berg, 2013), which interferes with α_2C_AR down-stream signaling (Cox et al., 2000; Trendelenburg et al., 2003b; Figure 1). Although α_2A_AR-mediated auto-inhibition was functional in female SHR, enhanced α_2C_AR signaling potentiated their effect also in this gender, as indicated by the elevated plasma norepinephrine and epinephrine concentrations observed after pre-treatment with ST-91+L-659,066 or losartan+L-659,066 compared to L-659,066 alone. A similar increase was not seen in the female WKY, or, previously, in male WKY (Berg, 2013). The reason for the potentiating effect of α_2C_AR on α_2A_AR auto-inhibition was not clear, but may be due to that α_2_AR were likely to form heterodimers (Figure 1), and the α_2A_α_2C_AR heterodimer was more stable against G-protein coupled receptor kinase 2 (GRK2) desensitization than the α_2A_α_2A_or α_2C_α_2C_ homodimers (Small et al., 2006). Another possible explanation in this *in vivo* model was that α_2C_AR may hamper renal renin release (Michel and Rump, 1996), and, in that manner, may lower AT1R-mediated counter-action of the α_2A∕2C_AR-mediated inhibition of release.

### The role of α_2_AR in control of vascular tension

One may assume that the VSMC AR are activated in a physiological situation primarily by sympathetic nerve transmitter release, whereas the endothelial cell receptors are influenced primarily by components present in the circulation. However, tyramine stimulates a massive and sustained release of norepinephrine. In this artificial situation, where norepinephrine NET re-uptake is prevented by the tyramine-stimulated release through the same transporter, the plasma concentration of norepinephrine is greatly increased, and the released norepinephrine may influence not only the VSMC receptors but also endothelial receptors. It is not possible to clearly differentiate between VSMC and endothelial activation in this *in vivo* experimental model, but vasodilatory components attributable to norepinephrine-activated endothelial receptors were not detected. However, one may assume that the tyramine-stimulated release of norepinephrine was much greater than that needed for VSMC activation. The impact of drugs on the tyramine-induced TPR-response was therefore likely to reflect a direct effect on the VSMC receptors rather than on the presynaptic receptors with subsequent differences in norepinephrine release. This conclusion was supported by the inverse relationship between plasma catecholamine levels and the TPR-response to tyramine, for instance in rats pre-treated with L-659,066. Tyramine, with an action restricted to peripheral, sympathetic nerve endings, therefore allowed an investigation of presynaptic control of norepinephrine release and, independently, the VSMC-response to the endogenously released norepinephrine.

In the 12–14 weeks-old female rats, clonidine and ST-91 induced a transient rise in TPR, like previously observed in male rats of the same age. In the males, the rise in TPR was reduced by L-659,066 (Berg et al., 2012), compatible with studies on genetically modified mice, where the initial clonidine-induced vasoconstriction was due to activation of VSMC α_2B_AR (Link et al., 1996). The vasoconstrictory TPR-response to clonidine was less in female SHR than in WKY, most likely due to a simultaneous clonidine-induced reduction in central sympathetic output in SHR. This conclusion was deduced from observations made in male rats, where clonidine induced a transient, L-659,066-sensitive reduction in HR in WKY, but a sustained, L-659,066-insensitive bradycardia and late vasodilatory TPR-response in SHR (Berg et al., 2012). A clonidine-induced, transient bradycardia was observed also in female WKY and a sustained bradycardia and late vasodilatation in female SHR, suggesting an elevated central sympathetic cardiovascular control in SHR also in this gender.

Also the α_2C_AR may be involved in the vasoconstrictory response to clonidine and ST-91, since an L-659,066-sensitive rise in TPR was seen in male rats also in response to an α_2C_-selective agonist, i.e., *m*-nitrobiphenyline (Berg, 2013), which in addition contained α_2A+B_AR antagonistic properties (Crassous et al., 2007). Although VSMC α_2C_AR did not contribute to BP-control in genetically modified mice (MacDonald et al., 1997), agonist-stimulated α_2C_AR-mediated vasoconstriction has been demonstrated in veins and arterioles (Chotani et al., 2004; Görnemann et al., 2007; Corboz et al., 2011). However, the initial rise in TPR in response to ST-91 was highly variable in the female rats, and the effect of L-659,066 on this response was therefore inconclusive. A similar variation was not observed in male rats (Berg, 2013). It may therefore be suggested that differences in the oestrous cycle and oestrogen-induced mobilization of α_2C_AR to VSMC surface, as demonstrated in human, cutaneous, arteriolar VSMC (Eid et al., 2007), caused differences in α_2C_AR-mediated vasoconstriction when stimulated with agonist. The VSMC α_2C_AR has been shown to be mobilized to the cell surface also by cold, and in that manner contribute to cold-induced vasoconstriction (Chotani et al., 2000). A possible influence of the oestrous cycle may be restricted to the VSMC since ST-91 alone had no effect on the tyramine-induced catecholamine overflow to plasma.

As in male WKY, the α_2_AR agonists clonidine and ST-91 had no effect on the TPR-response to the tyramine-induced norepinephrine release in female WKY. However, clonidine reduced the TPR-response in female SHR, but not in the male SHR (Berg and Jensen, 2013). The mechanism underlying the effect of clonidine in female SHR was not clear, but may result from stimulation of endothelial α_2_AR-mediated vasodilatation, as observed in male SHR under other conditions (Shafaroudi et al., 2005; Berg and Jensen, 2011). However, unlike that in the male SHR, where L-659,066 did not alter the TPR-response to tyramine (Berg and Jensen, 2013), L-659,066 eliminated the TPR-response to tyramine in female SHR. L-659,066 eliminated the TPR-response also in male WKY and even reversed the response to vasodilatation in the female WKY. Moreover, ST-91, which enhanced the effect of L-659,066 in male SHR, had no potentiating effect in female SHR. Thus, although the TPR-response to tyramine was higher and more sustained in female SHR than in female WKY, α_2_AR control of vascular tension was clearly more functional than in the male SHR, although not as effective as in the female WKY.

Unlike that in male WKY (Berg, 2013; Berg and Jensen, 2013), losartan abolished the L-659,066-dependent vasodilatory response to tyramine in the female WKY. The reason for this observation was not clear, but a similar effect was seen for the L-659,066-dependent increase in norepinephrine overflow, which was slightly less when combined with losartan. Although losartan restored the hampering effect of L-659,066 on tyramine-induced vasoconstriction in male SHR (Berg, 2013; Berg and Jensen, 2013), it had no additional effect on the inhibitory effect of L-659,066 on this response in female SHR. It therefore appeared that when the α_2_AR control of vascular tension was functional as in the female, AT1R activity had less potentiating effect on norepinephrine induced vasoconstriction, even though losartan clearly lowered TPR baseline also in the female SHR.

## Conclusions

These studies show that α_2_AR-mediated control of catecholamine release and vascular tension is functional in female, 12–14 weeks-old SHR, unlike that previously observed in male SHR of the same age. Functional α_2_AR-mediated control of catecholamine release may represent an important mechanism in protecting females against hypertension and delaying the development of disease in this gender.

## Funding

The Norwegian Council on Cardiovascular Diseases and Anders Jahres' Fond.

## Author contributions

The author confirms being the sole contributor of this work and approved it for publication.

### Conflict of interest statement

The author declares that the research was conducted in the absence of any commercial or financial relationships that could be construed as a potential conflict of interest.
